# Simultaneous Quantitation of Lipid Biomarkers for Inflammatory Bowel Disease Using LC–MS/MS

**DOI:** 10.3390/metabo11020106

**Published:** 2021-02-12

**Authors:** Yashpal S. Chhonker, Shrey Kanvinde, Rizwan Ahmad, Amar B. Singh, David Oupický, Daryl J. Murry

**Affiliations:** 1 Clinical Pharmacology Laboratory, Department of Pharmacy Practice and Science, College of Pharmacy, University of Nebraska Medical Center, Omaha, NE 68198, USA; y.chhonker@unmc.edu; 2 Center for Drug Delivery and Nanomedicine, Department of Pharmaceutical Sciences, College of Pharmacy, University of Nebraska Medical Center, Omaha, NE 68198, USA; shreykanvinde@gmail.com (S.K.); david.oupicky@unmc.edu (D.O.); 3 Department of Biochemistry and Molecular Biology, University of Nebraska Medical Center, Omaha, NE 68198, USA; rizwan.ahmad@unmc.edu (R.A.); amar.singh@unmc.edu (A.B.S.); 4 Fred and Pamela Buffett Cancer Center, University of Nebraska Medical Center, Omaha, NE 68198, USA; 5 VA Nebraska-Western Iowa Health Care System, Omaha, NE 68105, USA

**Keywords:** eicosanoids, LC–MS/MS, inflammatory bowel disease (IBD), biomarker, prostaglandins, colitis

## Abstract

Eicosanoids are key mediators and regulators of inflammation and oxidative stress that are often used as biomarkers for severity and therapeutic responses in various diseases. We here report a highly sensitive LC-MS/MS method for the simultaneous quantification of at least 66 key eicosanoids in a widely used murine model of colitis. Chromatographic separation was achieved with Shim-Pack XR-ODSIII, 150 × 2.00 mm, 2.2 µm. The mobile phase was operated in gradient conditions and consisted of acetonitrile and 0.1% acetic acid in water with a total flow of 0.37 mL/min. This method is sensitive, with a limit of quantification ranging from 0.01 to 1 ng/mL for the various analytes, has a large dynamic range (200 ng/mL), and a total run time of 25 min. The inter- and intraday accuracy (85–115%), precision (≥85%), and recovery (40–90%) met the acceptance criteria per the US Food and Drug Administration guidelines. This method was successfully applied to evaluate eicosanoid metabolites in mice subjected to colitis versus untreated, healthy control mice. In summary, we developed a highly sensitive and fast LC−MS/MS method that can be used to identify biomarkers for inflammation and potentially help in prognosis of the disease in inflammatory bowel disease (IBD) patients, including the response to therapy.

## 1. Introduction

Eicosanoids are major bioactive signaling mediators and regulators of inflammation and oxidative stress [1]. They are potential biomarkers for various disease states and pathological conditions, including inflammatory bowel disease (IBD) [2,3]. Eicosanoids are derived from dihomo-linolenic acid (DGLA), arachidonic acid (AA), and eicosapentaenoic acid (EPA) and formed via cyclooxygenase enzymes (COX), lipoxygenase enzymes (LOX), and cytochrome P450, as well as nonenzymatic oxidation [4,5]. Eicosanoids comprise over 100 lipid mediators, including prostaglandins (PG), thromboxanes (TX), leukotrienes (LT), hydroxyeicosatetraenoic acids (HETEs), dihydroxyeicosatetraenoic acids (DHETs), hydroxyeicosapentaenoic acids (HEPEs), lipoxins (LXs), resolvins (RvEs), and epoxyeicosatrienoic acids (EET) [3,6].

Eicosanoids play an important role in various physiological functions in the body [1]. Prostaglandin E2 (PGE2) regulates tumor angiogenesis in prostate cancer [7], whereas leukotrienes and lipoxins regulate vasoconstriction and vascular permeability [8,9]. Additionally, 20-hydroxyeicosatetraenoic acid (20-HETE) causes cerebral micro-vessel constriction [10], plays a role in cerebral blood flow autoregulation [11,12], and regulates new blood vessel growth [13]. Conversely, epoxyeicosanoid (EET) metabolites produce microvascular dilation [14,15], increase cerebral blood flow [16], and protect neurons and astrocytes from ischemic cell death in vitro [17,18]. Alterations in eicosanoid-generating enzymes have been identified during disease progression, including cardiovascular disease, stroke, myocardial infarction, asthma, Crohn’s disease, hypertension, gastric cancer, and colorectal cancer [19,20,21,22,23]. Given the clinical interest in eicosanoids and the complexity of their responses to biological stimuli, it is necessary to systematically evaluate their tissue-specific alterations during different disease states, as it may help evaluate disease progression and assess the benefit of specific therapeutic interventions.

A variety of analytical tools have been developed and validated for the separation, detection, and quantification of eicosanoids, including high-performance liquid chromatography (HPLC)-UV [24,25,26], enzyme immunoassays [27], LC–fluorescence detection [28], electrophoresis [29,30], immuno-affinity chromatography (IAC) [31], gas chromatography–mass spectrometry (GC-MS and GC–MS/MS) [32,33], and liquid chromatography–mass spectrometry (LC–MS/MS) [34,35,36,37,38,39]. The HPLC-UV methods to quantitate eicosanoids have limited sensitivity and typically require large sample volumes and long run times [24,25,26]. Immunoassays have been used for the quantification of eicosanoids; however, they are limited by cross-reactivity and low sensitivity [27]. Derivatization of samples and an analysis using LC–fluorescence improves on the sensitivity and specificity of UV detection; however, the process is time-consuming and is associated with the need for additional sample preparation [28]. The GC-MS/MS analysis provides a high sensitivity and resolution of the eicosanoids; however, the methods are associated with extensive sample preparation and the need for a derivatization of the sample analytes [32,33].

The development of a relatively sensitive and selective LC-MS/MS assay can overcome the above-mentioned limitations and offer a reliable method for the quantification of eicosanoids in various biological matrices [38,40,41,42,43]. We developed and validated a sensitive and selective LC–MS/MS method for the simultaneous quantification of 66 eicosanoids in colon samples derived from healthy mice and from mice with infectious colitis as a model for IBD. Notably, IBD is the chronic, remittent inflammation of the gastrointestinal tract (GIT) and, on a biochemical level, is characterized by the upregulation of proinflammatory cytokines, eicosanoids, and the subsequent infiltration of immune cells into the colonic tissue [44]. Additionally, targeting eicosanoid signaling has recently been identified as a potential therapeutic intervention in IBD [45]. Taken together, the utilization of a validated LC-MS/MS method to identify eicosanoid biomarkers for inflammation may help facilitate our understanding of the mechanisms involved in IBD disease progression and provide a quantitative method to assessing the impact of therapeutic intervention.

## 2. Results and Discussion

### 2.1. LC-MS/MS Method Development

A LC-MS/MS method for the quantitation of eicosanoids in mouse colons was developed and validated. All eicosanoids were analyzed in the negative-ionization mode, and each eicosanoid standard was optimized for multiple reaction monitoring (MRM), including the selection of MRM pairs for each eicosanoid (*m*/*z* values for precursor and product ions, respectively) and collision energies (Table 1). Mass spectrometer parameters were optimized during method development to the maximize sensitivity and selectivity.

**Figure 1:**
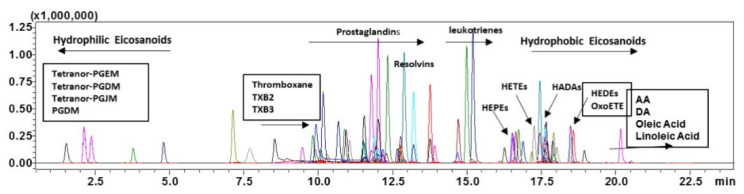
Representative overlaying chromatograms of all eicosanoid standards at 50 ng/mL under final chromatography and detection conditions. Thromboxane (TXB), hydroxy eicosapentaenoic acid (HEPEs), hydroxyeicosatetraenoic acid (HETEs), hydroxy docosahexaenoic Acid (HADAs), hydroxy eicosadienoic acid (HEDEs), oxo-eicosatetraenoic acid (OxoETE), Arachidonic acid (AA) and docosahexaenoic Acid (DA).

A representative LC-MS/MS overlaying the chromatograms of all eicosanoid standards is shown in Figure 1. Individual chromatograms are shown in Supplementary Materials Figure S1.

**Figure 2:**
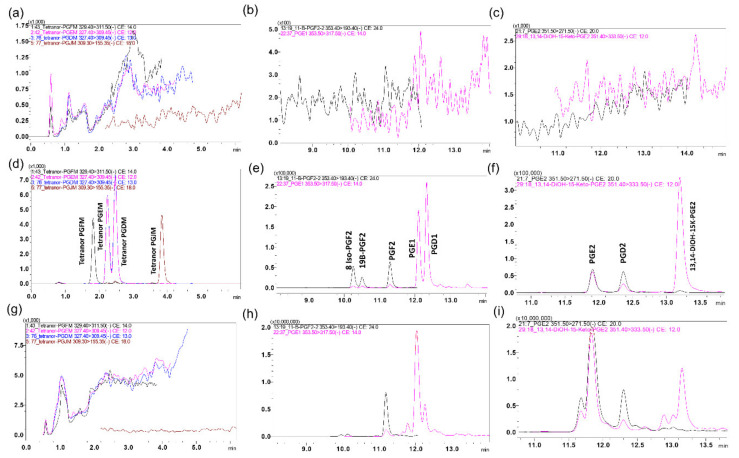
Representative overlaying multiple reaction monitoring (MRM) ion-chromatograms of isobaric eicosanoids that also share fragmentation patterns but were separated chromatographically: (**a**–**c**) blank charcoal-stripped surrogate matrix of selected analytes, (**d**–**f**) spiked in charcoal-stripped surrogate matrix of selected analytes (0.5 g/mL), and (**g**–**i**) healthy colon sample of selected analytes.

Eicosanoids represent a large family of endogenous compounds, and many members of this family are isobaric with related physiochemical properties, including isomers and stereoisomers. Many eicosanoids not only have the same mass but, also, have matching fragmentation patterns, resulting in the same MRM transitions. Moreover, many eicosanoids undergo insource fragmentation, which results in fragments with similar masses to other eicosanoids. In addition, interfering peaks may possibly arise from other unidentified endogenous components of the matrix. Therefore, MS/MS specificity by itself is not adequate to separate all eicosanoids, and chromatographic resolution is needed for their separation. For example, the isobaric compounds PGE2, PGD2, and 13,14-dihydro-15-k-PGE2 were identified through the same 351.40 > 333.50 MRM transition but were separated chromatographically. Similarly, the isobaric compounds PGF2a, 11-b PGF2a, and 8-iso-PGF2a were identified through the same 353.40 > 193.40 MRM transition but were separated chromatographically (Figure 2).

**Figure 3:**
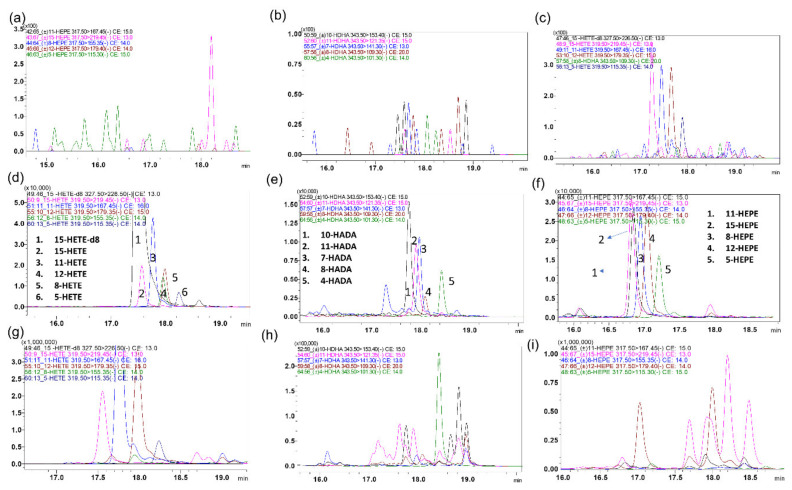
Representative overlay MRM ion-chromatograms of isobaric eicosanoids that share some fragments, as well as retention times, were distinguished via specific MRMs: (**a**–**c**) blank charcoal-stripped surrogate matrix of selected analytes, (**d**–**f**) spiked in charcoal-stripped surrogate matrix of selected analytes (0.5 g/mL), and (**g**–**i**) healthy colon sample of selected analytes.

In addition to chromatographic resolution, isobaric compounds such as HETEs, HEPEs, HEDEs, and HADAs, with similar MRMs, can be distinguished if they produce specific MRMs. For example, isobaric HETEs such as 5-HETE, 8-HETE, 11-HETE, 12-HETE, and 15-HETE were partially resolved chromatographically, but every isomer produced unique fragments, namely 319.50 > 115.35, 319.50 > 155.35, 319.50 > 167.45, 319.50 > 1179.35, and 319.50 > 219.45, respectively [46,47,48]. However, these specific MRMs were less sensitive than common MRMs such as 319.50 > 301.35. Similarly, the isobaric compounds such as 5-HEPE, 8-HEPE, 11-HEPE, 12-HEPE, and 15-HEPE produced unique fragments that were not produced by the other isomers (Figure 3).

The LC conditions were optimized to separate all eicosanoids of interest with a desired peak shape and signal intensity using a Shim-Pack XR-ODSIII, 150 × 2.00 mm, 2.2 µm column. The less hydrophobic eicosanoids, including PGs, TXS, and LTs eluted earlier, were not sensitive to changes in the mobile phase pH. In contrast, acidic mobile phases resulted in a better peak shape and longer retention of the more hydrophobic eicosanoids, including HETEs, HEPEs, HEDEs, HADAs, AA, and DA. Therefore, acetic acid was used as an aqueous and organic mobile phase modifier. Under our final LC-MS/MS conditions, all selected eicosanoids were resolved from each other in less than 25 min, and all standards produced single peaks. The exceptions were TXB2 and TXB3 and their d4-labeled ISs, each of which produced two peaks (completely chromatographically resolved) that belonged to their anomers. The anomers for both compounds were detected in standard, as well biological, samples.

In LC-MS analyses, it is critical to prepare calibration curves in similar or equivalent matrices to study samples due to matrix effects on the ionization of the analytes. This becomes problematic for endogenous analytes, including eicosanoids, where analyte-free blank matrices are not available to spike with analyte standards of known concentrations for the construction of calibration curves. Various approaches are followed to solve the endogenous background problem in blank matrices for the construction of calibration curves, including background subtraction [38,49], the standard addition method [50,51], matrix stripping with charcoal [52], and the use of surrogate matrices or stable isotope-labeled standards [53,54,55,56,57,58,59,60,61,62,63].

As discussed above, every method for the quantification of endogenous compounds has advantages and disadvantages. Therefore, we applied and compared the various approaches for the quantification of eicosanoids in a colon homogenate and found that the charcoal-stripped surrogate matrix was the most accurate and suitable method for this application. Most eicosanoids were completely depleted, but some eicosanoids with high endogenous levels had trace residual peaks in the homogenate after stripping with charcoal. For these eicosanoids, the background peak area of the remaining trace levels was subtracted from the peak area of the calibration curve standards, which allowed the construction of calibration curves with high accuracy and precision. Using analyte/IS peak area ratios, rather than absolute analyte peak areas, the recoveries of the eicosanoids in the charcoal-stripped matrix were similar to those in the unstripped homogenate (data not shown), which indicates that the matrix effect was the same for the study samples (unstripped colon homogenate) and calibration curve surrogate matrix.

Three calibration curves (CC) were used to cover all analytes in a colon homogenate at the relevant physiological concentrations, namely 0.01–200 ng/mL, 0.05–200 ng/mL, and 1–200 ng/mL. Different dynamic ranges were used, because the various eicosanoids had different sensitivity, endogenous concentrations, and/or signal linearity. For example, the lower limit of quantification (LLOQ) of AA and DA were 1 ng/mL in CC, not due to limitations in the detection sensitivity (limit of detection 0.01 ng/mL) but, rather, due to the relatively high background of these eicosanoids in the blank matrix used to construct the calibration curve, which did not allow a consistent subtraction from the peak areas of spiked standards below 1 ng/mL. We developed a specific, highly sensitive LC-MS/MS method and further validated its application for the simultaneous quantification of 66 eicosanoids in colon tissues. A summary of the current methods related to eicosanoids quantification in biomatrices by LC-MS/MS are shown in Supplementary Table S1. By comparison, our method resulted in an improved sensitivity and was linear over a large concentration range, required a small sample volume, and allowed the quantitation of 66 eicosanoids in 25 min. The addition of a solid phase extraction (SPE) resulted in an acceptable recovery and no matrix effect. Our validated method was accurate, precise, and sensitive, allowing for the routine analysis of eicosanoids in preclinical and clinical samples.

### 2.2. Method Validation

The method was validated for each analyte using calibration curves (*n* = 3) prepared on three consecutive days. Three dynamic ranges were used to cover all analytes at the relevant physiological concentrations, namely 0.01–200 ng/mL, 0.05–200 ng/mL, and 1–200 ng/mL. The method of charcoal stripping was used to prepare the calibration curve and quality control (QC) samples. A linear plot of the relation between the concentration and peak area ratio was fitted using weighted (1/C^2^) linear regression. Therefore, the differences in the lower limit of quantification of the various eicosanoids are not necessarily due to differences in the sensitivity of the analytes but, rather, due to the differences in the endogenous background levels in the blanks used for building the calibration curves. There were no endogenous interfering peaks identified at the retention times of the analytes of interest. The correlation coefficient was higher than 0.998 for all eicosanoids in the surrogate matrices, confirming the linearity of the assay in the selected calibration ranges. The analytes and IS peak retention time were stable, with a relative standard deviation (%RSD) within the acceptable limit of ±5%. There was no significant peak (>20% of the LLOQ) in zero samples injected after the high-quality control (HQC) samples, indicating no carryover effect.

Intraday and inter-day accuracy and precision were determined to evaluate the reliability and reproducibility of this method. The inter-day accuracy and precision of the standards prepared in the colon homogenate are shown in Table 2. The accuracy was ≤80% at the LLOQ and ≤85% at the other four QC concentrations for all eicosanoids in the surrogate matrix. The precision (%RSD) was ≤20% at the LLOQ and ≤15% at the other four QC. These data confirm that the method described has a satisfactory accuracy and precision for the quantitation of all the analytes of interest.

The precision for the dilution integrity of 1:5 and 1:10 dilutions was within the acceptable limits for all analytes, which are within the acceptance limits of ±15% for precision (CV) and 85–115% for accuracy. The results suggested that plasma samples with concentrations above the upper limit of quantitation can be determined by the appropriate dilution.

### 2.3. Recovery and Matrix Effect

Several protein precipitations and SPE methods were investigated to increase the extraction recovery and decrease matrix effect. The large variations in the physicochemical properties between the different classes of eicosanoids resulted in different extraction recoveries of these compounds. The average extraction recovery of all the analytes ranged from 40% to 90% in the surrogate matrix (data not shown). Moreover, the extraction recoveries of six ISs were constant, ranging from 40% to 90%, with a CV% of <10% in both the surrogate matrix and colon homogenate (Supplementary Table S2). Although a higher recovery is better for most analytes, this simple SPE could completely satisfy the accurate quantitation for all the eicosanoids (Table 2). We found similar recoveries in the surrogate matrix and colon homogenate (data not shown).

Furthermore, the matrix effect was negligible and well within the acceptable range (±15%) in both the surrogate and colon homogenate (Supplementary Table S2). Additionally, the SPE treatment removed most of interfering proteins, which also contributed to the minimal matrix effect (ME) observed.

### 2.4. Stability Studies

The stability of eicosanoids in the stocks and colon homogenate were studied under various conditions, as outlined in Section 3.7. Instability was defined as a 20% or more decrease in the peak area compared to freshly prepared QCs under different conditions (Supplementary Materials Table S3). All eicosanoids were stable in the stock solutions and the colon homogenate at 4 °C up to 24 h in the auto-sampler, except for compounds AA, AA-d8, and 12-OxoETE. The eicosanoids were also stable in the colon homogenate at room temperature on the bench up to two hours, except for LTD4, LTC4 and 14,15-LTC4, 11-De TXB3, and 12-OxoETE. In addition, after four hours, a few more compounds were found to be unstable at room temperature (Supplementary Table S3). To avoid this, sample processing was always performed on wet ice. All eicosanoids were stable under these processing conditions. After 30 days of long-term storage, all eicosanoids were stable in the surrogate matrix, except for LTD4, LTC4, PGE3, 14,15-LTC4, 11-De TXB3, and 12-OxoETE.

### 2.5. Application to Metabolomics of Data Profiling

**Figure 4:**
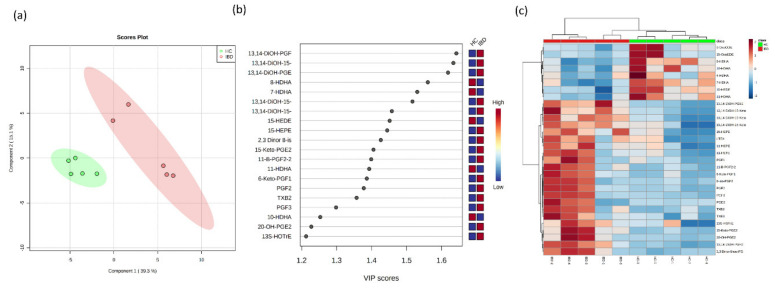
Multivariate Partial Least Squares-Discriminate Analysis (PLS-DA) analyses of the healthy control (HC) and inflammatory bowel disease (IBD) colon samples. (**a**) Two-dimensional PLS-DA score plot of the HC (green) and IBD (red) are separated from the other groups, indicating very distinct lipid metabolite compositions. Component 1 indicates the degree of variation between the groups based on their total metabolite contents, and component 2 indicates the differences within the groups. It can be noted that the component 1 value is three-fold greater than component 2, indicating that the differences between the groups are greater than within the groups. (**b**) Important features identified by the variable importance in the projections (VIP) scores; metabolites (20) with a VIP >1 have an above-average influence. (**c**) Hierarchical clustering analysis (heatmap) for the 30 most-altered metabolites. Each column in the heatmap represents a replicate, and each row represents a metabolite. The red and blue colors indicate the relative abundance intensity of each metabolite within a sample.

Out of the 66 evaluated eicosanoids, 62 eicosanoids and their metabolites were detected above the LLOQ in all samples (Supplementary Table S4). The metabolite concentrations were divided into colitis (IBD) and healthy control (HC) groups, and a multivariate supervised Partial Least Squares-Discriminate Analysis (PLS-DA) was used on the transformed metabolomic dataset to identify the overall differences between the groups. First, the PLS-DA was applied to compare the metabolic concentrations in the HC with those of the IBD colon samples. As shown in the score plot (Figure 4a), the samples from the HCs were largely separated from the colitis group. The differences between the groups were greater than the differences within the groups. The variable importance in the projections (VIPs) of all the peaks from the PLS-DA models were taken as the coefficients for the metabolite selection, and the variables with VIP values >1 were considered to be contributors to the group discrimination [41]. The metabolites with the highest VIP scores and their respective concentrations in the colon samples are shown in Figure 4b. A hierarchical clustering analysis (heatmap) was generated to visualize the relative abundance of the serum eicosanoids in the healthy control (red) and IBD groups (green) (Figure 4c). Overall, clear differences were observed between the HC and IBD for 30 eicosanoids, allowing for discrimination between the two groups.

**Figure 5:**
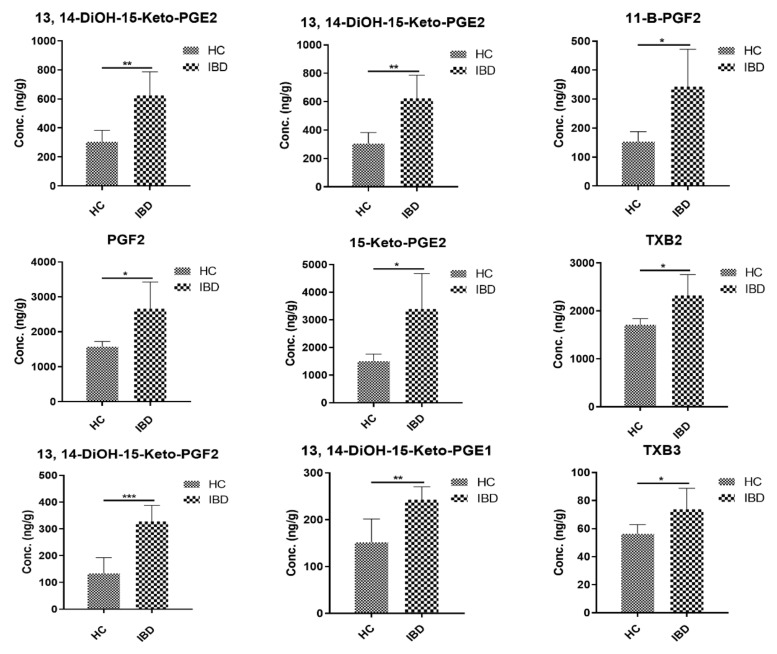
Eicosanoid concentrations in healthy controls (HC) and IBD colon samples. Significance was assessed with the *t*-test, and all *p*-values were <0.05 (mean ± SD, *n* = 5).

The COX and LOX pathways predominantly metabolize AA. The COX pathway results in the formation of stable PGs, TXs, and prostacyclin, while the LOX pathway leads to HPETEs, HETEs, and LTs [64,65]. PGs and TXs are very potent mediators of inflammation and play a very important role in proinflammatory, as well as anti-inflammatory, processes [66]. Several reports have shown an upregulation in the levels of PGs and TXs in IBD, especially during relapse [67,68]. In agreement with these previous reports, our study revealed an upregulation in the levels of several prostaglandins (PGs). Prostaglandin E family (PGEs) and prostaglandin F family (PGFs) (commonly known as the primary PGs) were the first two active PGs that were first identified for their roles in inflammation [69]. We observed an approximately two-fold increase in the colonic levels of PGE2 and its various metabolites (Figure 5). Concentrations of 13,14-DiOH-15-Keto-PGE2 and 15-Keto-PGE2 were increased two-fold in IBD mice compared to HC. Few studies have indicated the therapeutic benefits of targeting 15-Keto-PGE2 to treat sepsis, which is a systemic inflammation [70]. PGE2 is one of the most abundantly produced PGs across species. It plays an important role in the maintenance of many biological functions, including immune regulation, blood pressure regulation, and GI barrier integrity. It is especially of interest in inflammation, as it is involved in modulating processes resulting in defining the signs of inflammation, including redness, swelling, and pain [1]. It is also involved in the activation of pain neurons and the resulting responses. A knockout mouse model of mPGES-1 has been extensively used to study the role of PGE2 in inflammation [71]. Another role of PGE2 is the production of interleukin 17 (IL-17), which has been shown to be involved in the development and progression of IBD [72]. Targeting PGE2 by gene knockout studies has been proposed as an anti-inflammatory strategy in order to circumvent nonsteroidal anti-inflammatory drug (NSAID) associated toxicity. As a result, PGE2 antagonists are being evaluated for anti-inflammatory therapies [73,74]. Elevations in the levels of PGF2α and its metabolites have also been reported in patients with arthritis [75].

We also found elevated concentrations of PGF2α and its metabolite 8-iso-PGF2α in colitis-affected mice, which is in accordance with previous reports [76,77]. The levels of 11-β PGF2 (PGD2 metabolite) were increased 2.2-fold in IBD mice. PGD2 metabolites have been associated with mast cell activation, and it has been reported as a marker in allergen-induced asthma [78,79] It has also been reported to be an anti-inflammatory signal in lung inflammation and arthritis [78]. Further, an increase by ~1.5-fold in the TXB2 and TXB3 levels has also been observed in IBD group as compared to HC. TXs have been shown to be upregulated in the IBD [80]. Thromboxane synthase is the enzyme responsible for the generation of thromboxanes. The colonic immunohistochemistry of colon specimens obtained from IBD patients revealed that a higher percentage of cells in the lamina propria showed staining for thromboxane synthase, indicating its possible role in mucosal inflammation [81]. Ridrogel and R68070 are thromboxane synthase inhibitors that have been implicated to show therapeutic benefits in the treatment of IBD [82]. An increase in the PG and TX levels has been shown to have a direct relationship with inflammatory disease activity [83]. Looking at the byproducts of the lipoxygenase pathway, we found a two-fold increase in the level of LTE4. LTE4 has been implicated to play a role in many inflammatory conditions [84,85], including IBD. There are multiple reports supporting the quantification of urinary LTE4 as a noninvasive biomarker for the assessment of IBD activity [86,87]. The LTB4 and 5-HETE colonic levels have been shown to be increased in human IBD; however, we did not observe any significant changes in the concentrations of either eicosanoids. Collectively, the concentrations of PGs and TXs exhibited the greatest differences between HC and mice with IBD. The developed LC-MS/MS method was used to quantify the colonic and plasma concentrations of eicosanoids in HC and IBD mice. The development of sensitive and specific methods to evaluate the differences in inflammatory biomarkers in a disease can aid in identifying the disease severity and impact of a therapy on the disease progression.

## 3. Materials and Methods

### 3.1. Chemicals and Reagents

All unlabeled chemical standards (Supplementary Table S5) and stable deuterated isotope-labeled internal standards (IS), including PGE2 -d4, TXB2-d4, AA-d8, 15-HETE-d8, LTB4-d8, and Resolvin D1-d5, were purchased from Cayman Chemicals (Ann Arbor, MI, USA). LC-MS grade methanol (MeOH), acetonitrile (ACN), formic acid, and acetic acid were obtained from Fisher Scientific (Fair Lawn, NJ, USA). Oasis HLB SPE cartridges (60 mg/3 mL) were from Waters Corporation (Milford, MA, USA). Centrifuge tube filters were from Corning Co. (Corning, NY, USA). Ultrapure water was obtained from a water purification system (ThermoFisher Scientific, NJ). All other chemical reagents from Sigma (St. Louis, MO, USA).

### 3.2. Liquid Chromatographic and Mass Spectrometric Conditions

A Shimadzu Nexera UPLC system equipped with two pumps (LC-30 AD) and a column oven (CTO-30AS), along with an auto-sampler (SIL-30AC), were used (Shimadzu Scientific Instruments, Columbia, MD). Mass spectrometric detection was performed on an LC-MS/MS 8060 system (Shimadzu Scientific Instruments, Columbia, MD, USA) equipped with a DUIS source operated in negative-electrospray ionization mode. The MS/MS system was operated at unit resolution in the multiple reaction monitoring (MRM) mode. All chromatographic separations were performed with a Shim-Pack XR-ODSIII, 150 × 2.00 mm, 2.2 µm as the analytical columns equipped with a Shim-Pack C_18_ guard column (Shimadzu Scientific Instruments, Columbia, MD, USA).

The mobile phase comprised of 0.1% acetic acid in water (mobile phase A) and acetonitrile (ACN) (mobile phase B) at total flow rate of 0.37 mL/min. The chromatographic separation was achieved using 25-min gradient elution (Table 3). The injection volume was kept at 20 μL of all samples.

The compound-dependent mass spectrometer parameters, such as temperature, voltage, gas pressure, etc., were optimized by auto-method optimization via a precursor ion search for each analyte and the internal standard (IS) using a 1-µg/mL solution in methanol. All eicosanoids were detected in the negative-ionization mode with the following instrument-dependent mass spectrometer parameters, such as nebulizer gas: 2.2 L/min, heating gas: 10 L/min, drying gas: 10 L/min, interface temperature: 300 °C, desolvation line temperature: 250 °C, heat block temperature: 400 °C, and interface temperature: 300 °C. The multiple reaction monitoring (MRM) transitions for each analyte and IS, as well as their respective optimum MS parameters, such as the declustering potential (DP) and collision energy (CE), are shown in Table 1. The UPLC and MS systems were controlled by LabSolutions LCMS Ver.5.6 software (Shimadzu Scientific Inc., Columbia, MD, USA).

### 3.3. Preparation of Stock, Calibration Standard, and Quality Control Sample Preparation

Aliquots from the original stock solutions (each analyte) were mixed to prepare a spiking solution and stored at −70 °C until use. The calibration curves of the various eicosanoids were divided into three ranges based on the sensitivity or endogenous concentrations expected and included the following: 0.01–200 ng/mL, 0.05–200 ng/mL, and 1–200 ng/mL (Table 1). The charcoal-stripped blank surrogate matrix (200 µL) was spiked with mixed spiking analyte (10×) solutions to get a final standard concentration, 20 µL each, and vortexed for 30 s. Samples were then extracted as described below. Six stable-labeled eicosanoids were used as the IS (20 µL) at a final concentration of 50 ng/mL. Quality control (QCs) samples were prepared in five replicates at four different concentrations: the lower limit of quantification (LLOQ), low-quality control (LQC), middle-quality control (MQC), and high-quality control (HQC) (Table 2), independent of the calibration standards.

### 3.4. Sample Preparation

All analytes were extracted from CC and QC samples in the surrogate matrix [88] and colon samples by the solid phase extraction (SPE) method using Oasis^®^ HLB 3 cc/60-mg SPE cartridges (Waters Corporation, Milford, MA, USA). Each colon sample was accurately weighed and then homogenized with triple-distilled deionized water at a 7-fold dilution factor using a TissueLyserII (Qiagen Science Germantown, MD, USA). A colon homogenate sample (200 μL) was spiked with IS (20 μL) and diluted with 5% acetic acid in water (1500 μL), vortexed for 30 s, and then loaded onto SPE cartridges preconditioned with MeOH (2 mL), followed by 0.1% acetic acid in water (2 mL). Loaded cartridges were washed with 5% MeOH (2 mL) and eluted with 100% MeOH (2 mL). Eluates for all standards and samples were evaporated under vacuum at room temperature and reconstituted with 50% ACN in water (100 μL).

### 3.5. Method Validation

The developed LC-MS/MS method for eicosanoid quantitation was validated according to the US Food and Drug Administration (FDA) guidelines for industry bioanalytical method validation [89]. The sensitivity of the method was ascertained from the signal-to-noise ratio (S/N) of the response of the analyte in the calibration standards and was required to be greater than 3 for the limit of detection (LOD) and greater than 10 for the LLOQ. The calibration curves were plotted between the peak area ratio (analyte/IS) and concentration for all analytes.

Intra- and inter-day accuracy and precision were evaluated from replicate analyses (*n* = 5) of QC samples containing analytes at variable concentrations (LLOQ, LQC, MQC, and HQC). The precision was calculated in terms of the % of the relative standard deviation (%RSD). The accuracy was expressed as % Bias (Equation (1)). The acceptance criteria of the data included accuracy within ± 15% standard deviations (SD) from the nominal values and precision within ± 15% RSD, except for the LLOQ, which was within ± 20% for accuracy and precision.

% Bias = (observed concentration − nominal concentration) × 100/nominal concentration (1)

The carryover was checked by injecting two zero samples straight after a HQC sample. The first zero sample response was required to be <20% of the processed LLOQ sample response.

A dilution effect was investigated to ensure that the sample could be diluted with water without affecting the concentration. Analyte-spiked surrogate matrices prepared at 2000-ng/mL concentrations were diluted with stripped surrogate matrices at dilution factors of 5 and 10 in five replicates and analyzed. As part of the validation, five replicates had to meet both a precision of ≤15% and accuracy of 100% ± 15% criteria.

### 3.6. Extraction Recovery and Matrix Effect

Recoveries of analytes and labeled ISs from a charcoal-stripped surrogate matrix were determined by dividing the peak area ratio of the analyte to IS (after subtracting the endogenous background, if any) from before the extraction spiked blank samples to those from neat unextracted standards for both the low and high QCs (*n* = 3), along with the. recovery of stable isotope ISs into a colon homogenate, spiking ISs before and after the extraction, respectively, and comparing the resultant peak areas.

The matrix effect (ME) was evaluated by spiking stable isotope ISs (40 and 150 ng/mL) into the surrogate matrix and colon homogenate and comparing the peak areas with spiked reconstitution solvent samples in five replicates. The corresponding peak areas of the ISs in both spiked post-extractions (A) were then compared with those of the reconstitution solvent samples (B) at equivalent concentrations. The ratio (A/B × 100) was defined as the ME. The matrix effects in these two matrices were considered similar if the corresponding peak areas in the surrogate matrix and untreated homogenate did not deviate more than 20%.

### 3.7. Stability Studies

Stability experiments were carried out to examine the analyte stability in stock solutions and colon homogenate at different storage conditions. Stability studies included auto-sampler stability (at 4 °C for 24 h), bench-top stability (at room temperature for 6 h), freeze-thaw stability (three freeze-thaw cycles), and long-term stability (at −80 °C for 30 days) for both the low and high QCs (*n* = 3).

### 3.8. Animal Handling, Disease Induction, and Tissue Collection

C57BL/6 mice (6–8 weeks old) were obtained from Charles River Laboratories (Wilmington, MA, USA). All animal experiments were conducted using the protocol approved by the University of Nebraska Medical Center Institutional Animal Care and Use Committee. The *Citrobacter rodentium* (*C. rodentium*)-induced model of colitis was used to induce chronic colonic inflammation in mice, as previously described [90,91]. In brief, the microbial culture of *C. rodentium* was delivered to mice (*n* = 5/group; oral gavage) in a 100-µL volume containing 5 × 10^8^ colony-forming units of bacteria. The healthy control (HC) group (*n* = 5) was administered 100-µL Luria broth. Fourteen days after the bacterial gavage, mice were sacrificed. The colon was harvested from both the HC and IBD groups, cleaned of fecal contents, frozen, and stored at −80 °C for further analysis.

### 3.9. Metabolomics Data Processing

The raw data obtained were converted to concentration data (ng/gL) using Lab Solution software, version 5.8 (Shimadzu Scientific, Inc., Columbia, MD, USA). Metabolomic data analysis was performed using the MetaboAnalyst 4.0 platform [92]. Data processing started with a data integrity check, missing or zero values, filtering, normalization, and then generalized log transformation. Data analysis included a fold change, *t*-test, heatmap, and multivariate analyses, including the Principal Component Analysis (PCA) and Partial Least Squares-Discriminate Analysis (PLS-DA). Data divided several variables of the metabolomic profile into compounds for data reduction using the PCA; this aimed at summarizing the data into much fewer variables called scores, which were weighted averages of the original variables. PLS-D allowed the selection of molecules with a VIP index higher than 1.0 as potential biomarkers.

## 4. Conclusions

In summary, we developed a highly sensitive and specific LC-MS/MS method and further validated its application for the simultaneous quantification of 66 eicosanoids in colon tissues. This analytical method is sensitive, selective, and accurate for the characterization of detailed eicosanoids profiles in colonic tissues and plasma from mice with intestinal inflammation. The LLOQ of this LC-MS/MS method was sufficient to accurately determine the low-abundance eicosanoid metabolites. This method was successfully applied to evaluate the eicosanoid metabolites in mice subjected to colitis versus untreated healthy control mice. The mouse model used in this study, *C. rodentium*, has been widely used for colitis studies and shown to induce reproducible and robust inflammatory responses. The described LC-MS/MS method in this study will facilitate a better understanding of the eicosanoid pathological and physiological roles and further help to design future studies to assess biomarkers to predict efficacy, toxicity, and clinical outcomes in the therapeutic regimen/strategy to treat IBD.

## Figures and Tables

**Table 1:** Summary of multiple reaction monitoring (MRM) (*m*/*z*), retention time (RT), Q1 pre-rod potential, collision energy (CE), Q3 pre-rod potential, and dynamic range of all 66 eicosanoids and internal standards (IS).

Analytes ID	Analytes	MRM (*m*/*z*)	RT (Min.)	Q1 Pre Vias (V)	CE (V)	Q3 Pre Vias (V)	Dynamic Range (ng/mL)
1	PGJ2	333.50 > 233.40	13.7	12	10	15	0.01–200
3	20-OH-PGE2	367.40 > 287.45	4.9	12	17	19	0.01–200
4	PGB2	333.50 > 235.45	13.9	11	19	15	0.01–200
6	PGD2	351.50 > 271.50	12.0	12	17	17	0.01–200
7	PGE2	351.50 > 271.50	11.6	12	17	17	0.01–200
8	AA	303.50 > 259.50	20.6	22	14	11	1–200
9	15-HETE	319.50 > 219.45	17.3	11	14	13	0.01–200
10	12-HETE	319.50 > 179.35	17.7	11	15	10	0.01–200
11	11-HETE	319.50 > 167.45	17.5	11	16	10	0.01–200
12	8-HETE	319.50 > 155.35	17.7	16	14	24	0.01–200
13	5-HETE	319.50 > 115.35	18.0	11	14	16	0.01–200
14	LTE4	438.40 > 235.45	12.8	10	23	14	1–200
15	LTD4	495.50 > 177.35	11.0	11	20	10	1–200
16	LTC4	624.50 > 272.40	12.0	22	24	12	1–200
17	LTB4	335.50 > 195.40	14.7	12	16	11	0.01–200
18	13,14-DiOH-15-Keto-PGE2	351.40 > 333.50	12.9	11	12	10	0.01–200
19	11-B-PGF2	353.40 > 193.40	10.1	12	24	12	0.01–200
20	8-iso-PGF2	353.40 > 193.40	9.8	12	24	12	0.01–200
21	PGF2	353.40 > 193.40	10.9	12	24	12	0.01–200
22	15-Keto-PGE2	349.30 > 235.40	12.3	18	15	15	0.01–200
23	6-Keto-PGF1	369.40 > 245.45	7.7	12	25	15	0.01–200
24	TXB2	369.50 > 169.40	10.0	13	18	10	0.01–200
29	13,14-DiOH-PGF2	355.40 > 311.50	12.0	11	25	14	0.01–200
30	PGF1	355.40 > 311.50	11.0	11	25	14	0.01–200
31	13,14-DiOH-15-Keto-PGF2	353.50 > 113.40	12.7	12	25	11	0.01–200
32	13,14-DiOH-15-Keto-PGE1	353.40 > 335.50	13.2	12	13	22	0.01–200
33	PGD1	353.50 > 317.50	12.1	12	17	15	0.01–200
34	13,14-DiOH-PGE1	355.30 > 337.55	12.4	11	16	11	0.01–200
35	TXB3	367.50 > 169.35	8.6	10	17	10	0.01–200
36	15-deoxy-delta 12,14 PGJ2	315.50 > 271.50	13.8	12	14	17	0.01–200
37	PGE1	353.50 > 317.50	11.8	12	17	15	0.01–200
38	PGE3	349.50 > 269.50	10.2	12	16	17	0.01–200
39	PGD3	349.50 > 269.50	10.7	12	16	17	0.01–200
40	PGF3	351.50 > 307.50	9.5	12	18	14	0.1–200
41	14,15-LTC4	624.50 > 272.40	13.0	22	23	18	1–200
42	Tetranor-PGEM	327.40 > 309.45	2.2	11	12	21	0.1–200
43	Tetranor-PGFM	329.40 > 311.50	1.8	16	14	10	0.1–200
44	11-De TXB3	365.40 > 303.50	10.4	13	16	20	0.01–200
45	2,3 Dinor 8-iso PGF2	325.50 > 237.50	7.2	12	13	15	0.01–200
53	Docosahexaenoic Acid	327.50 > 283.55	20.3	11	12	18	1–200
54	9(10)-DiHOME	313.50 > 201.40	15.2	11	22	12	0.01–200
55	12(13)-DiHOME	313.50 > 183.40	15.1	11	22	11	0.01–200
56	4-HDHA	343.50 > 101.30	18.1	11	14	29	0.01–200
57	7-HDHA	343.50 > 141.30	17.7	12	13	23	0.01–200
58	8-HDHA	343.50 > 109.30	17.8	11	20	30	0.01–200
59	10-HDHA	343.50 > 153.40	17.5	12	15	25	0.01–200
60	11-HDHA	343.50 > 121.35	17.7	12	15	18	0.01–200
61	11-HEDE	323.40 > 199.45	18.5	11	21	12	0.01–200
62	15-HEDE	323.40 > 223.50	18.6	11	20	14	0.01–200
63	5-HEPE	317.50 > 115.30	17.0	12	15	18	0.01–200
64	8-HEPE	317.50 > 155.35	16.7	10	14	26	0.01–200
65	11-HEPE	317.50 > 167.45	16.6	11	15	10	0.01–200
66	12-HEPE	317.50 > 179.40	16.8	12	14	10	0.01–200
67	15-HEPE	317.50 > 219.45	16.7	12	13	13	0.01–200
68	9(S)-HOTrE	293.50 > 171.35	16.2	12	15	10	0.01–200
69	13(S)-HOTrE	293.50 > 224.45	16.3	11	14	13	0.01–200
70	5-OxoETE	317.50 > 203.40	18.6	10	18	12	0.01–200
71	12-OxoETE	317.50 > 153.40	18.0	16	16	25	0.01–200
72	15-OxoETE or 15-KETE	317.50 > 113.35	17.7	12	20	18	0.01–200
73	9-OxoODE or 9-KEDE	293.50 > 185.40	17.8	11	21	11	0.01–200
74	15-OxoEDE or 15-KEDE	321.50 > 223.45	19.1	11	23	14	0.01–200
76	Tetranor-PGDM	327.40 > 309.45	2.8	11	13	20	0.1–200
77	Tetranor-PGJM	309.30 > 155.35	4.2	12	22	24	0.01–200
78	Resolvin D1	375.60 > 141.30	12.9	10	15	23	1–200
79	Resolvin D2	375.60 > 175.35	12.2	10	23	10	0.1–200
80	Resolvin D3	375.60 > 147.40	12.0	10	22	23	0.1–200
25	TXB2-d4	373.50 > 173.40	10.0	13	19	10	NA
26	PGE2-d4	355.50 > 275.55	11.5	13	17	18	NA
27	AA-d8	311.40 > 267.55	20.6	10	13	17	NA
46	15 -HETE-d8	327.50 > 226.50	17.2	16	13	14	NA
47	LTB4-d4	339.50 > 197.45	14.7	10	16	12	NA
81	Resolvin D1-d5	380.50 > 141.30	12.8	13	16	22	NA

NA: Not Applicable.

**Table 2:** Accuracy and precision of eicosanoids in the charcoalated surrogate matrix.

Analytes ID		LLOQ (0.01 ng/mL)		LQC (0.05 ng/mL)		MQC (40 ng/mL)		HQC (150 ng/mL)	
		Accuracy	%RSD	Accuracy	%RSD	Accuracy	%RSD	Accuracy	%RSD
1	PGJ2	108.9	14.3	106.6	2.6	103.7	4.0	86.9	3.9
3	20-OH-PGE2	112.6	5.1	92.4	5.5	104.0	6.2	108.9	0.5
4	PGB2	114.9	4.9	99.7	9.1	101.7	4.9	86.8	1.9
6	PGD2	103.9	11.2	108.7	3.2	92.5	2.8	90.6	4.9
7	PGE2	99.2	10.0	98.1	1.8	97.8	5.6	87.3	3.7
9	15-HETE	100.7	17.3	112.7	3.8	96.7	6.0	85.3	1.3
10	12-HETE	104.6	5.4	108.4	4.8	96.9	1.4	87.5	3.7
11	11-HETE	104.5	5.6	114.5	3.2	87.1	1.4	91.1	3.3
12	8-HETE	190.5	14.8	114.7	4.3	92.1	5.5	84.7	5.8
13	5-HETE	109.9	14.3	110.4	3.4	103.1	2.9	87.0	0.9
17	LTB4	112.5	18.4	110.1	6.6	92.0	5.9	103.8	6.1
18	13,14-DiOH-15-Keto-PGE2	102.3	11.2	107.2	3.2	97.8	2.5	90.2	2.2
19	11-B-PGF2	107.9	18.2	106.7	1.4	105.8	4.3	87.3	0.8
20	8-iso-PGF2	112.2	9.1	103.3	4.5	103.9	5.6	88.3	2.3
21	PGF2	110.2	9.1	104.0	4.5	100.6	4.3	88.0	2.1
22	15-Keto-PGE2	101.3	11.2	107.5	2.7	101.9	2.5	91.0	2.8
23	6-Keto-PGF1	90.9	19.2	92.0	6.1	107.6	5.1	107.7	1.6
24	TXB2	109.6	15.7	108.0	4.6	90.1	5.1	86.5	2.5
29	13,14-DiOH-PGF2	107.3	5.4	109.9	8.9	104.7	4.3	107.8	1.7
30	PGF1	90.1	19.2	106.1	8.9	101.5	3.1	89.1	2.9
31	13,14-DiOH-15-Keto-PGF2	116.6	10.2	105.7	5.1	101.4	4.2	88.7	3.4
32	13,14-DiOH-15-Keto-PGE1	110.8	9.1	99.9	5.5	102.5	3.8	95.7	3.7
33	PGD1	101.2	10.0	115.1	3.2	92.2	4.1	88.1	3.2
34	13,14-DiOH-PGE1	107.7	5.4	109.6	4.7	100.5	4.4	90.9	3.0
35	TXB3	91.3	11.1	98.6	3.7	93.2	3.0	105.5	3.8
36	15-deoxy-delta 12,14 PGJ2	98.4	15.8	85.6	16.1	100.9	2.8	88.0	3.1
37	PGE1	86.0	18.3	104.8	3.9	100.2	3.8	87.2	3.2
38	PGE3	99.0	6.0	105.8	6.7	99.4	2.5	85.6	1.6
39	PGD3	90.4	3.1	99.8	2.3	97.3	0.2	93.0	2.2
44	11-De TXB3	85.6	6.9	104.2	9.8	96.7	2.6	91.3	1.1
45	2,3 Dinor 8-iso PGF2	107.4	9.1	95.1	2.5	102.8	6.6	102.3	2.1
54	9(10)-DiHOME	109.6	9.1	113.2	2.6	90.7	4.7	86.8	1.4
55	12(13)-DiHOME	112.1	18.4	112.3	2.2	100.6	2.0	91.7	5.7
56	4-HDHA	119.4	4.7	104.2	7.4	105.2	6.1	86.9	1.0
57	7-HDHA	112.9	15.7	107.3	16.5	85.1	9.6	86.0	4.8
58	8-HDHA	118.1	16.4	104.9	7.3	103.8	5.4	94.9	3.2
59	10-HDHA	118.4	18.9	112.0	3.6	89.3	4.1	85.9	5.5
60	11-HDHA	109.3	21.7	104.5	5.9	103.7	5.6	91.1	1.6
61	11-HEDE	107.1	14.8	109.5	9.1	87.3	3.0	84.2	8.0
62	15-HEDE	114.6	5.1	109.2	3.3	92.0	5.4	86.6	5.3
63	5-HEPE	107.2	19.5	107.5	5.7	110.8	3.0	88.6	1.2
64	8-HEPE	100.2	20.0	114.9	4.0	101.6	11.5	91.7	2.1
65	11-HEPE	118.1	4.9	106.3	9.4	89.1	3.3	87.4	5.6
66	12-HEPE	119.1	4.7	107.9	4.0	105.7	3.4	88.0	3.2
67	15-HEPE	109.1	15.7	111.0	4.1	92.2	5.9	85.6	2.6
68	9(S)-HOTrE	110.3	18.2	109.4	7.2	111.6	7.4	85.9	1.1
69	13(S)-HOTrE	105.4	19.5	111.1	1.4	108.2	4.5	86.7	3.8
70	5-OxoETE	99.2	5.6	98.8	4.5	105.8	8.1	90.1	1.4
71	12-OxoETE	104.2	20.2	107.4	9.1	88.7	4.3	86.0	18.8
72	15-OxoETE or 15-KETE	120.0	8.3	114.7	8.7	96.5	3.7	85.3	4.7
73	9-OxoODE or 9-KEDE	122.2	4.7	112.9	2.5	104.5	5.0	89.3	4.6
74	15-OxoEDE or 15-KEDE	103.8	20.1	104.8	3.1	100.3	3.1	89.7	3.8
77	Tetranor-PGJM	83.9	6.7	103.0	4.2	113.3	6.2	114.9	3.2
78	Resolvin D1	108.7	19.5	103.7	14.0	104.6	3.5	89.7	2.6
		LLOQ (0.05 ng/mL)		LQC (2 ng/mL)		MQC (40 ng/mL)		HQC (150 ng/mL)	
40	PGF3	99.2	14.1	99.6	6.6	103.7	6.6	102.4	1.7
42	Tetranor-PGEM	120.4	1.0	111.7	14.2	89.5	12.3	96.7	2.4
43	Tetranor-PGFM	109.8	20.1	85.9	4.2	99.3	3.5	100.5	6.3
76	Tetranor-PGDM	98.5	6.3	86.4	5.0	113.8	8.0	114.7	2.7
79	Resolvin D2	105.5	15.4	102.6	12.3	96.0	6.7	89.1	0.3
80	Resolvin D3	106.7	11.4	85.7	3.5	91.1	9.0	86.3	12.6
		LLOQ (1 ng/mL)		LQC (2 ng/mL)		MQC (40 ng/mL)		HQC (150 ng/mL)	
8	AA	83.7	4.1	106.0	15.0	103.2	15.2	89.2	3.2
53	Docosahexaenoic Acid	114.2	5.6	111.5	12.2	94.5	12.2	85.0	10.8
14	LTE4	109.7	8.4	112.9	1.7	90.1	1.7	92.9	2.5
15	LTD4	112.2	9.9	85.2	1.7	93.1	1.7	112.0	1.8
16	LTC4	114.6	11.4	91.5	4.9	93.4	4.9	114.3	0.2
41	14,15-LTC4	98.8	9.1	86.4	5.0	90.2	5.0	112.6	3.1

**Table 3:** Linear gradient chromatographic method.

Time (Min)	% of Aqueous Phase (A)	% of Organic Phase (B)	Flow Rate (mL/Min)
0	80	20	0.37
10	60	40	0.37
20	0	100	0.37
23	0	100	0.37
23.1	80	20	0.37
25	80	20	0.37

## Data Availability

The data presented in this study are available in the article and in the Supplementary Materials.

## Supplementary Materials

The following are available online at https://www.mdpi.com/2218-1989/11/2/106/s1: Figure S1: Representative individual MRM ion-chromatograms of all eicosanoid standards at 50 ng/mL in charcoalated human plasma under final chromatography and detection conditions. The target compound is noted by an asterisk (*). Table S1: A list of all compounds and their chemical name, abbreviation, and CAS number. Table S2: Mean extraction recoveries and matrix effect of the ISs in the surrogate matrix and colon homogenate. Table S3: Eicosanoid stability in plasma under various storage conditions (Instability was defined as a 20% or more decrease in the peak under different storage conditions compared to freshly prepared QCs.). Table S4: Eicosanoid concentrations (ng/g) in the colons of healthy, as well as mice with C. rodentium-induced inflammation (mean ± SD, *n* = 5). Table S5: Summary of the comparison of this method with previously reported LC-MS/MS methods related to eicosanoid quantification in rat/mice plasmas and tissues.
