# Supplementary material for: Simultaneous Quantitation of Lipid Biomarkers for Inflammatory Bowel Disease Using LC–MS/MS
Source: Metabolites. 2021 Feb 12;11(2):106. doi: 10.3390/metabo11020106 (PMC7918109; doi:10.3390/metabo11020106)
Supplement: Supplementary file 1 [file metabolites-11-00106-s001.pdf]

Supplementary Materials

Simultaneous quantitation of lipid biomarkers for inflammatory bowel disease using LC–MS/MS

Yashpal S. Chhonker [1], Shrey Kanvinde [2], Rizwan Ahmad [3], Amar B. Singh [3,4,5], David Oupický [2,4], and Daryl J. Murry [1,4,*]

[1] Clinical Pharmacology Laboratory, Department of Pharmacy Practice and Science, College of Pharmacy, University of Nebraska Medical Center, Omaha, NE 68198, USA, y.chhonker@unmc.edu ; dj.murry@unmc.edu

[2] Center for Drug Delivery and Nanomedicine, Department of Pharmaceutical Sciences, College of Pharmacy, University of Nebraska Medical Center, Omaha, NE 68198, USA, shreykanvinde@gmail.com (S.K.); david.oupicky@unmc.edu (D.O.)

[3] Department of Biochemistry and Molecular Biology, University of Nebraska Medical Center, Omaha, NE 68198, USA, rizwan.ahmad@unmc.edu

[4] Fred and Pamela Buffett Cancer Center, University of Nebraska Medical Center, Omaha, NE 68198, USA, amar.singh@unmc.edu

[5] VA Nebraska-Western Iowa Health Care System, Omaha, NE 68105, USA

* Correspondence: dj.murry@unmc.edu; Tel.: +402-559-3790 or +402-559-2430

Table S1. Summarizes the comparison of this method with previously reported LC-MS/MS methods related to eicosanoid quantification in in rat/mouse plasma and tissues.

Method	No. of Eicosanoids quantified	Matrix	Matrix Volume	LOQ Ranges	Analysis run time (min)	Sample pretreatment	Reference
LC-MS/MS	23	Rat kidney tissue	100 mg	5.5 to 223.8pg	60	SPE	[1]
LC-MS/MS	137	Mouse tissue	Whole organ tissues	0.5 to 50 pg	20.1	SPE	[2]
LC-MS/MS	40	Rat tissue		0.01 to 0.1 nmol/g	30	SPE	[3]
LC-MS-MS	11	Rat brain tissue	Whole brain	0.10 ng/mL	12	SPE	[4]
LC-MS/MS	24	Mouse colon tissue	Whole colon	LOQ: 0.98 to 31 ng/mL	8.5	SPE	[5]
UPLC-MS/MS	48	Mouse serum and tissue samples	150 µL serum and 5 mg tissue	0.3 to 4.0 nM	8	SPE	[6]
LC-MS/MS	19	Rat brain tissue	20-40 mg	2.0 pg to 20 ng	35	SPE	[7]
UPLC-MS/MS	18	Rat brain tissue	1.5-2 gm	NA	27	SPE	[8]
UPLC-MS/MS	66	Mouse colon tissue	250uL homogenate (~50mg)	0.01 to 1.0 ng/mL	25	SPE	Current method

Table S2. Mean extraction recoveries and matrix effect of the ISs in surrogate matrix and colon homogenate.

Bio-Matrix	Analytes	Estimates (Mean ± SD, n=3)			
		Mean Extraction Recovery (%)		Absolute Matrix effect (%)	
		LQC	HQC	LQC	HQC
Surrogate Matrix	TXB2-d4	87.8 ± 1.3	86.4 ± 1.3	99.1 ± 6.2	85.2 ± 3
	PGE2-d4	90.4 ± 3.2	87.3 ± 3.3	102.3 ± 8.2	102 ± 2
	AA-d8	60.9 ± 5.8	68.5 ± 1.5	88.7 ± 2.5	89.5 ± 5.1
	15 -HETE-d8	49.9 ± 0.7	51.8 ± 2.5	102.1 ± 9.1	86.9 ± 4.2
	LTB4-d4	63.1 ± 2.6	66.1 ± 1.8	106.2 ± 9.4	90.7 ± 8.4
	Resolvin D1-d5	85.9 ± 1.4	78.3 ± 2.4	100.5 ± 2.5	88.9 ± 4.6
Mouse Colon homogenate	TXB2-d4	85.8 ± 1.4	83.1 ± 3	97.5 ± 5.7	84.2 ± 3
	PGE2-d4	84.7 ± 3	86.7 ± 4.9	93.5 ± 3.8	102 ± 2
	AA-d8	66.1 ± 4.5	62.9 ± 5.2	86.2 ± 3.3	89.5 ± 5.1
	15 -HETE-d8	48 ± 5.5	53.8 ± 8.2	95 ± 9.8	86.9 ± 4.2
	LTB4-d4	61.2 ± 1.7	54.9 ± 3.6	91.2 ± 12.6	90.7 ± 8.4
	Resolvin D1-d5	80.3 ± 2.2	71.2 ± 5.3	86 ± 1.5	88.9 ± 4.6

Table S3. Eicosanoids stability in plasma under various storage conditions. (Instability was defined as a 20% or more decrease in peak under different storage condition compare to freshly prepared QCs.)

Analytes ID	Analytes	Auto sampler at 4 °C for 24hr	Benchtop at 25 °C for 1 hr	Benchtop at 25 °C for 4hr	Freeze-thaw at -80 °C for three cycle	Long term at -80 °C for 30 days
1	PGJ2	89.4	105.8	108.4	99.3	87.0
3	20-OH-PGE2	89.3	98.7	90.4	89.2	93.9
4	PGB2	90.7	93.9	92.9	85.0	87.4
6	PGD2	92.3	81.1	89.6	81.3	83.3
7	PGE2	98.5	98.7	93.1	100.1	102.3
8	AA	59.2	109.2	88.0	110.0	91.1
9	15-HETE	94.2	108.8	108.0	105.8	100.8
10	12-HETE	91.8	101.5	110.3	94.9	95.3
11	11-HETE	92.1	107.7	107.9	104.3	100.1
12	8-HETE	93.6	101.6	123.4	96.0	96.1
13	5-HETE	95.3	104.2	107.3	101.9	97.5
14	LTE4	85.0	106.8	109.0	101.0	108.4
15	LTD4	102.2	54.7	5.5	35.0	78.9
16	LTC4	86.1	7.9	1.3	1.6	14.4
17	LTB4	89.3	89.5	84.5	79.6	81.1
18	13,14-DiOH-15-Keto-PGE2	87.0	81.4	46.8	66.2	78.0

19	11-B-PGF2	85.7	98.6	96.8	91.3	88.9
20	8-iso-PGF2	93.4	98.3	94.6	92.1	93.5
21	PGF2	104.9	103.5	102.0	104.9	107.5
22	15-Keto-PGE2	86.5	80.8	49.5	71.6	81.4
23	6-Keto-PGF1	94.3	98.6	97.1	97.8	98.2
24	TXB2	91.3	102.0	92.9	90.9	94.2
29	13,14-DiOH-PGF2	84.8	92.3	88.9	86.4	86.7
30	PGF1	94.4	99.6	94.7	96.1	96.1
31	13,14-DiOH-15-Keto-PGF2	100.1	95.5	95.5	95.1	96.4
32	13,14-DiOH-15-Keto-PGE1	97.0	89.6	79.5	89.0	94.4
33	PGD1	92.5	82.7	49.3	84.6	85.8
34	13,14-DiOH-PGE1	84.7	82.3	67.7	81.0	78.9
35	TXB3	102.6	100.1	93.4	97.1	101.2
36	15-deoxy-delta 12,14 PGJ2	95.8	91.9	78.7	86.6	92.0
37	PGE1	98.4	96.8	93.3	97.8	97.1
38	PGE3	85.4	91.0	75.9	71.0	69.5
39	PGD3	103.0	86.1	42.6	92.6	116.7
40	PGF3	101.8	108.0	101.7	103.3	99.3
41	14,15-LTC4	82.8	11.6	0.8	2.6	23.6
42	Tetranor-PGEM	93.9	95.4	72.7	71.4	91.1

43	Tetranor-PGFM	93.2	93.3	89.0	88.9	89.5
44	11-De TXB3	85.7	45.4	4.3	21.2	47.5
45	2,3 Dinor 8-iso PGF2	94.2	98.5	98.5	97.3	98.4
53	Docosahexaenoic Acid	98.3	107.8	88.5	108.1	91.9
54	9(10)-DiHOME	92.3	88.8	88.1	81.8	83.7
55	12(13)-DiHOME	95.1	91.3	86.8	83.7	84.5
56	4-HDHA	91.7	105.9	125.9	101.1	94.4
57	7-HDHA	93.9	95.6	101.2	84.2	92.5
58	8-HDHA	90.5	96.6	111.8	86.6	90.8
59	10-HDHA	92.9	97.6	101.7	90.5	94.6
60	11-HDHA	88.8	93.7	101.3	85.0	90.9
61	11-HEDE	100.9	96.0	98.9	86.5	93.8
62	15-HEDE	100.6	97.3	101.3	91.8	98.0
63	5-HEPE	93.7	95.9	115.8	86.1	94.0
64	8-HEPE	92.3	91.6	81.2	81.0	88.6
65	11-HEPE	99.4	92.2	96.2	88.2	92.4
66	12-HEPE	98.2	88.9	95.8	83.8	85.2
67	15-HEPE	94.0	92.4	100.5	92.0	91.7
68	9(S)-HOTrE	96.5	86.4	94.4	81.1	82.2
69	13(S)-HOTrE	92.0	98.0	106.3	98.0	100.2

70	5-OxoETE	91.7	93.7	95.0	86.8	90.6
71	12-OxoETE	75.5	61.2	25.1	47.6	57.1
72	15-OxoETE or 15-KETE	95.9	100.4	99.5	90.6	97.0
73	9-OxoODE or 9-KEDE	92.8	95.6	99.2	85.7	89.9
74	15-OxoEDE or 15-KEDE	91.2	93.4	92.0	86.4	90.3
76	Tetranor-PGDM	92.8	93.9	75.9	77.7	88.6
77	Tetranor-PGJM	93.5	95.3	89.4	90.5	93.9
78	Resolvin D1	95.7	92.2	86.2	88.2	92.6
79	Resolvin D2	84.8	93.8	91.8	92.0	89.6
80	Resolvin D3	98.1	98.7	98.4	100.9	100.1
25	TXB2-d4	97.1	98.9	90.3	93.6	96.0
26	PGE2-d4	95.8	91.9	88.7	86.6	92.0
27	AA-d8	71.2	109.5	81.3	99.3	83.2
46	15 -HETE-d8	94.8	101.4	92.8	96.1	98.0
47	LTB4-d4	86.8	87.3	80.1	85.7	87.7
81	Resolvin D1-d5	93.6	94.2	86.5	88.9	90.9

Table S4. Eicosanoids concentration (ng/g) in colon of healthy as well as mice having C. rodentium induced inflammation (mean ± SD, n=5).

Analyte ID	Analytes	Healthy Control (HC) colon Mean Conc (ng/g)	SD	IBD colon Mean conc ng/g)	SD
1	PGJ2	232.95	104.07	209.35	97.40
3	20-OH-PGE2	5.46	0.54	10.54	4.57
4	PGB2	28.00	16.11	41.93	28.92
6	PGD2	1169.16	279.36	1591.71	736.29
7	PGE2	5403.96	126.38	7159.45	2659.66
8	AA	86405.26	40368.75	89152.24	25309.67
9	15-HETE	1505.19	894.43	1910.82	654.19
10	12-HETE	3567.75	1974.72	4426.39	997.65
11	11-HETE	1820.55	518.63	3020.57	984.28
12	8-HETE	87.05	24.18	73.83	16.99
13	5-HETE	472.65	200.51	425.71	136.48
14	LTE4	14.29	7.83	26.44	8.20
15	LTD4	2.13	2.01	1.97	1.26
16	LTC4	82.41	32.92	95.17	17.84
17	LTB4	16.13	7.58	20.42	3.34
18	13,14-DiOH-15-Keto-PGE2	304.19	78.50	623.46	163.15

19	11-B-PGF2-2	153.09	34.06	343.30	128.43
20	8-iso-PGF2	93.03	7.28	139.43	47.09
21	PGF2	1573.86	147.54	2656.65	768.61
22	15-Keto-PGE2	1500.58	259.15	3394.34	1282.99
23	6-Keto-PGF1	11156.37	1458.98	17290.82	4123.40
24	TXB2	1709.22	129.25	2320.30	437.64
29	13,14-DiOH-PGF2	8.65	1.11	18.10	3.89
30	PGF1	448.30	63.46	604.04	164.68
31	13,14-DiOH-15-Keto-PGF2	133.54	59.29	327.53	60.56
32	13,14-DiOH-15-Keto-PGE1	151.70	50.06	242.49	28.08
33	PGD1	172.56	50.46	189.23	44.09
34	13,14-DiOH-PGE1	12.85	3.75	38.39	10.98
35	TXB3	56.27	6.55	73.85	14.93
36	15-deoxy-delta 12,14 PGJ2	474.16	144.32	514.20	243.44
37	PGE1	1651.08	115.04	1632.90	555.48
38	PGE3	75.04	9.52	100.61	41.26
39	PGD3	10.46	4.81	12.85	4.57
40	PGF3	28.99	3.08	55.00	21.01
41	14,15-LTC4	8.13	3.04	7.76	2.30
42	Tetranor-PGEM	BLQ		BLQ	

43	Tetranor-PGFM	BLQ		BLQ	
44	11-De TXB3	8.82	8.01	4.74	1.57
45	2,3 Dinor 8-iso PGF2	0.26	0.08	1.01	0.49
53	Docosahexaenoic Acid	24098.92	4739.54	29856.62	9036.01
54	(±)9(10)-DiHOME	702.59	195.61	783.14	222.86
55	(±)12(13)-DiHOME	757.36	243.15	839.03	227.31
56	(±)4-HDHA	163.08	78.86	96.55	42.28
57	(±)7-HDHA	15.46	2.09	9.48	2.56
58	(±)8-HDHA	51.16	10.31	27.51	6.66
59	(±)10-HDHA	27.17	6.35	18.63	3.91
60	(±)11-HDHA	31.18	7.21	19.01	4.65
61	(±)11-HEDE	355.45	163.41	394.61	115.52
62	(±)15-HEDE	225.39	42.92	137.29	36.45
63	(±)5-HEPE	15.74	5.36	11.91	2.37
64	(±)8-HEPE	44.91	45.50	33.68	15.17
65	(±)11-HEPE	93.53	42.65	160.43	47.15
66	(±)12-HEPE	418.97	196.19	518.99	161.69
67	(±)15-HEPE	32.46	27.97	93.81	28.16
68	9(S)-HOTrE	299.92	112.12	219.79	60.23
69	13(S)-HOTrE	268.19	129.65	445.28	100.18

70	5-OxoETE	784.73	880.14	270.66	126.57
71	12-OxoETE	511.97	515.20	722.71	245.14
72	15-OxoETE or 15-KETE	511.97	515.20	722.71	245.14
73	9-OxoODE or 9-KEDE	5175.68	2274.35	2952.75	697.94
74	15-OxoEDE or 15-KEDE	58.84	34.33	31.73	5.60
76	tetranor-PGDM	BLQ		BLQ	
77	tetranor-PGJM	BLQ		BLQ	
78	Resolvin D1	1.96	1.71	2.92	1.06
79	Resolvin D2	1.33	0.35	1.45	0.72
80	Resolvin D3	0.40	0.12	0.42	0.28

Table S5. List of all compounds their chemical name, abbreviation, and CAS No.

Analytes ID	Chemical Name	abbreviation	CAS No.
1	Prostaglandin J2	PGJ2	60203-57-8
3	20-hydroxy prostaglandin E2	20-OH-PGE2	57930-95-7
4	Prostaglandin B2	PGB2	13367-85-6
6	Prostaglandin D2	PGD2	41598-07-6
7	Prostaglandin E2	PGE2	363-24-6
8	Arachidonic acid	AA	506-32-1
9	15-hydroxyeicosatetraenoic acid	15-HETE	71030-36-9
10	12-hydroxyeicosatetraenoic acid	12-HETE	71030-37-0
11	11-hydroxyeicosatetraenoic acid	11-HETE	73804-65-6
12	8-hydroxyeicosatetraenoic acid	8-HETE	79495-84-4
13	5-hydroxyeicosatetraenoic acid	5-HETE	73307-52-5
14	Leukotriene E4	LTE4	75715-89-8
15	Leukotriene D4	LTD4	73836-78-9
16	Leukotriene C4	LTC4	72025-60-6
17	Leukotriene B4	LTB4	71160-24-2
18	13,14-dihydro-15-keto-prostaglandin E2	13,14-DiOH-15-Keto-PGE2	363-23-5
19	11-Beta prostaglandin F2α	11-B-PGF2	38432-87-0
20	8-iso prostaglandin F2α	8-iso-PGF2	177020-26-7

21	Prostaglandin F2α	PGF2	4510-16-1
22	15-keto-prostaglandin E2	15-Keto-PGE2	26441-05-4
23	6-keto-prostaglandin F1α	6-Keto-PGF1	58962-34-8
24	Thromboxane B2	TXB2	54397-85-2
29	13,14-dihydro-prostaglandin F2α	13,14-DiOH-PGF2	27376-74-5
30	Prostaglandin F1α	PGF1	10164-73-5
31	13,14-dihydro-15-keto-prostaglandin F2α	13,14-DiOH-15-Keto-PGF2	27376-76-7
32	13,14-dihydro-15-keto-prostaglandin E1	13,14-DiOH-15-Keto-PGE1	5094-14-4
33	Prostaglandin D1	PGD1	17968-82-0
34	13,14-dihydro-prostaglandin E1	13,14-DiOH-PGE1	19313-28-1
35	Thromboxane B3	TXB3	71953-80-5
36	15- deoxy-delta 12,14 Prostaglandin J2	15-deoxy-delta 12,14 PGJ2	87893-55-8
37	Prostaglandin E1	PGE1	745-65-3
38	Prostaglandin E3	PGE3	802-31-3
39	Prostaglandin D3	PGD3	71902-47-1
40	Prostaglandin F3α	PGF3	802-31-3
41	13,14-leukotriene C4	14,15-LTC4	75290-60-7
42	Tetranor-prostaglandin E metabolite	Tetranor-PGEM	24769-56-0
43	Tetranor-prostaglandin F metabolite	Tetranor-PGFM	23109-94-6
44	11-dehydro-thromboxane B3	11-De TXB3	67910-12-7

45	2,3-dinor-8-iso prostaglandin F2α	2,3 Dinor 8-iso PGF2	221664-05-7
53	Docosahexaenoic Acid	Docosahexaenoic Acid	6217-54-5
54	(±)9,10-dihydroxy-12Z-octadecenoic acid	9(10)-DiHOME	263399-34-4
55	(±)12,13-dihydroxy-9Z-octadecenoic acid	12(13)-DiHOME	263399-35-5
56	4-hydroxy Docosahexaenoic Acid	4-HDHA	90906-40-4
57	7-hydroxy Docosahexaenoic Acid	7-HDHA	90780-55-5
58	8-hydroxy Docosahexaenoic Acid	8-HDHA	90780-54-4
59	10-hydroxy Docosahexaenoic Acid	10-HDHA	90780-50-0
60	11-hydroxy Docosahexaenoic Acid	11-HDHA	87018-59-5
61	(±)-11-hydroxy-12E,14Z-eicosadienoic acid	11-HEDE	5598-37-8
62	(±)-15-hydroxy-12E,14Z-eicosadienoic acid	15-HEDE	77159-57-0
63	(±)-5-hydroxy-6E,8Z,11Z,14Z,17Z-eicosapentaenoic acid	5-HEPE	83952-40-3
64	(±)-8-hydroxy-6E,8Z,11Z,14Z,17Z-eicosapentaenoic acid	8-HEPE	99217-77-3
65	(±)-11-hydroxy-6E,8Z,11Z,14Z,17Z-eicosapentaenoic acid	11-HEPE	99217-78-4
66	(±)-12-hydroxy-6E,8Z,11Z,14Z,17Z-eicosapentaenoic acid	12-HEPE	81187-21-5
67	(±)-15-hydroxy-6E,8Z,11Z,14Z,17Z-eicosapentaenoic acid	15-HEPE	88852-33-9
68	9S-hydroxy-10E,12Z,15Z-octadecatrienoic acid	9(S)-HOTrE	89886-42-0
69	13S-hydroxy-10E,12Z,15Z-octadecatrienoic acid	13(S)-HOTrE	87984-82-5
70	5-oxo-6E,8Z,11Z,14Z-eicosatetraenoic acid	5-OxoETE	106154-18-1
71	12-oxo-6E,8Z,11Z,14Z-eicosatetraenoic acid	12-OxoETE	108437-64-5

72	15-oxo-5Z,8Z,11Z,13E-eicosatetraenoic acid	15-OxoETE or 15-KETE	81416-72-0
73	9-oxo-10E,12Z-octadecadienoic acid	9-OxoODE or 9-KEDE	54232-59-6
74	15-oxo-11Z,13E-eicosadienoic acid	15-OxoEDE or 15-KEDE	105835-44-7
76	9α-hydroxy-11,15-dioxo-13,14-dihydro-2,3,4,5-tetranor-prostan-1,20-dioic acid	Tetranor-PGDM	70803-91-7
77	8-((1R,2S)-2-(2-carboxyethyl)-5-oxocyclopent-3-en-1-yl)-6-oxooctanoic acid	Tetranor-PGJM	1352751-83-7
78	Resolvin D1	Resolvin D1	872993-05-0
79	Resolvin D2	Resolvin D2	810668-37-2
80	Resolvin D3	Resolvin D3	916888-47-6
25	Thromboxane B2-d4	TXB2-d4	1346112-79-5
26	Prostaglandin E2-d4	PGE2-d4	34210-10-1
27	Arachidonic acid-d8	AA-d8	69254-37-1
46	15-hydroxyeicosatetraenoic acid-d8	15 -HETE-d8	84807-87-4
47	Leukotriene B4-d4	LTB4-d4	124629-74-9
81	Resolvin D1-d5	Resolvin D1-d5	1881277-32-2

Supplementary Figure S1. Representative individual MRM ion-chromatograms of all eicosanoid standards at 50 ng/ml in surrogate matrix under final chromatography and detection conditions. Target compound is noted by asterisk (*).

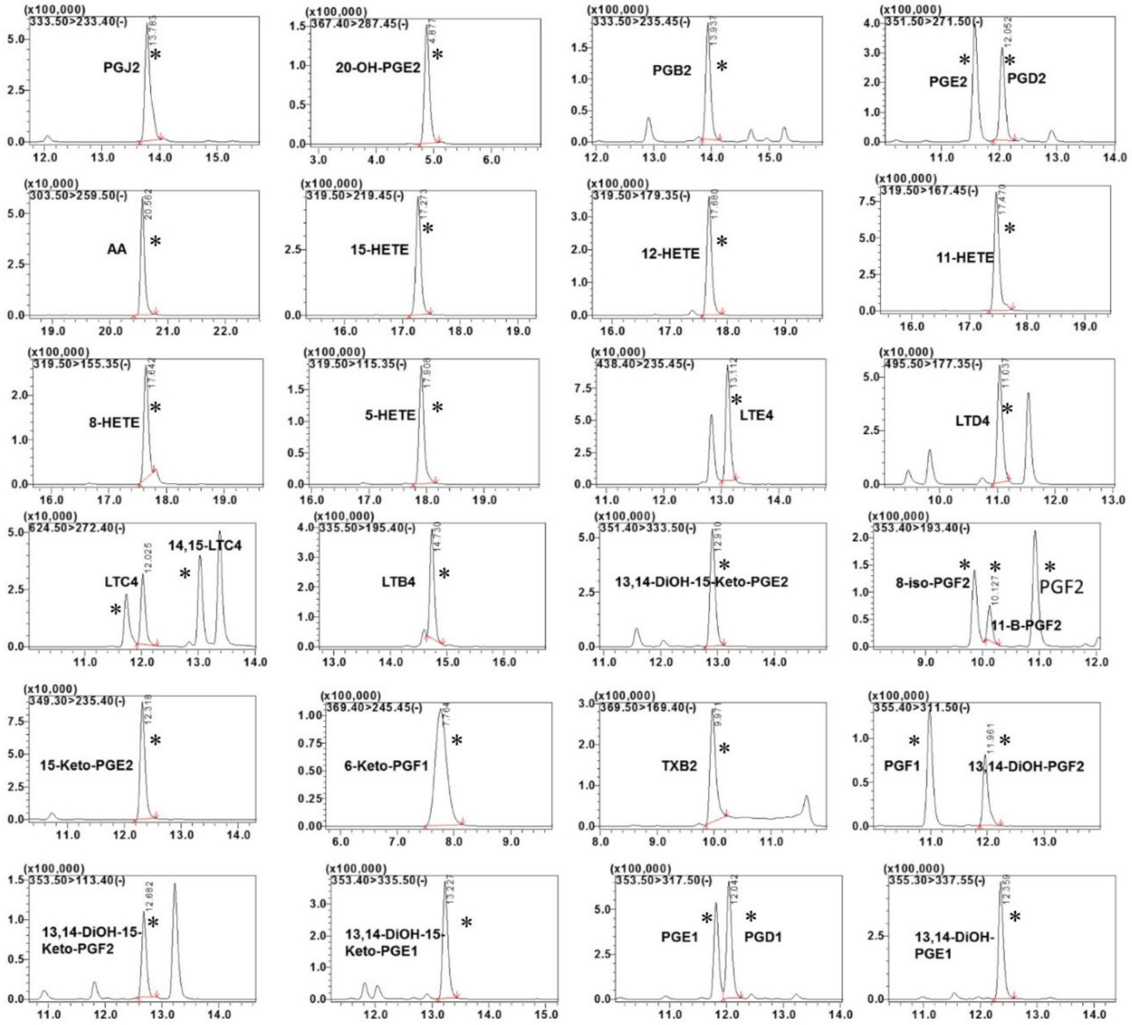

Figure S1. Continued….

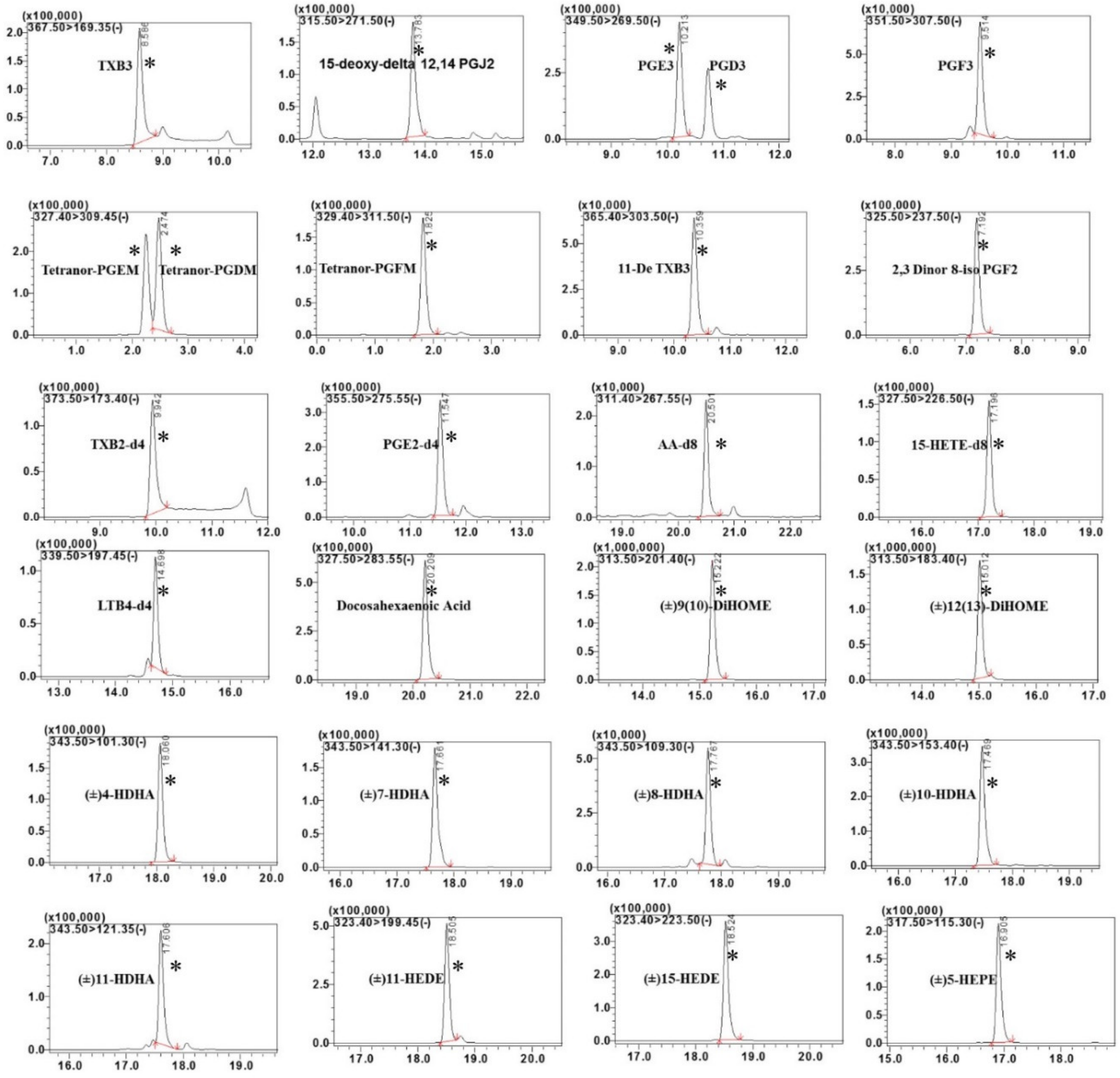

Figure S1. Continued….

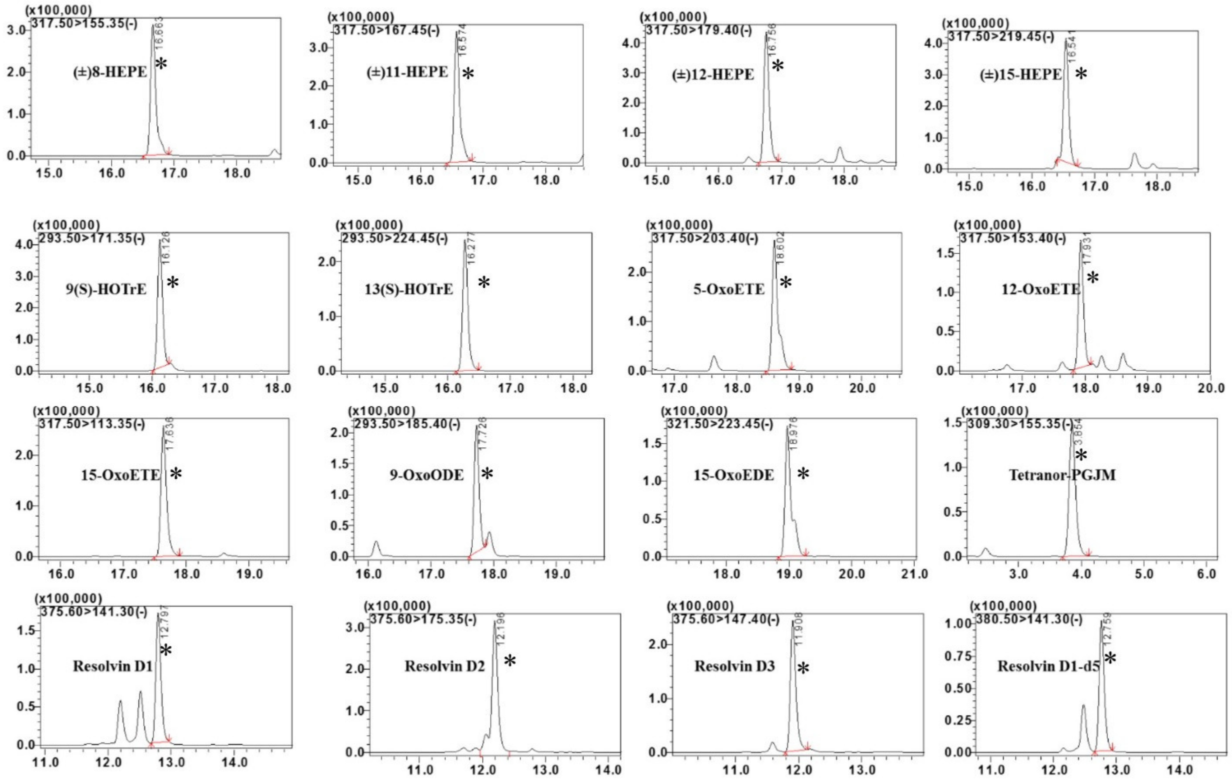

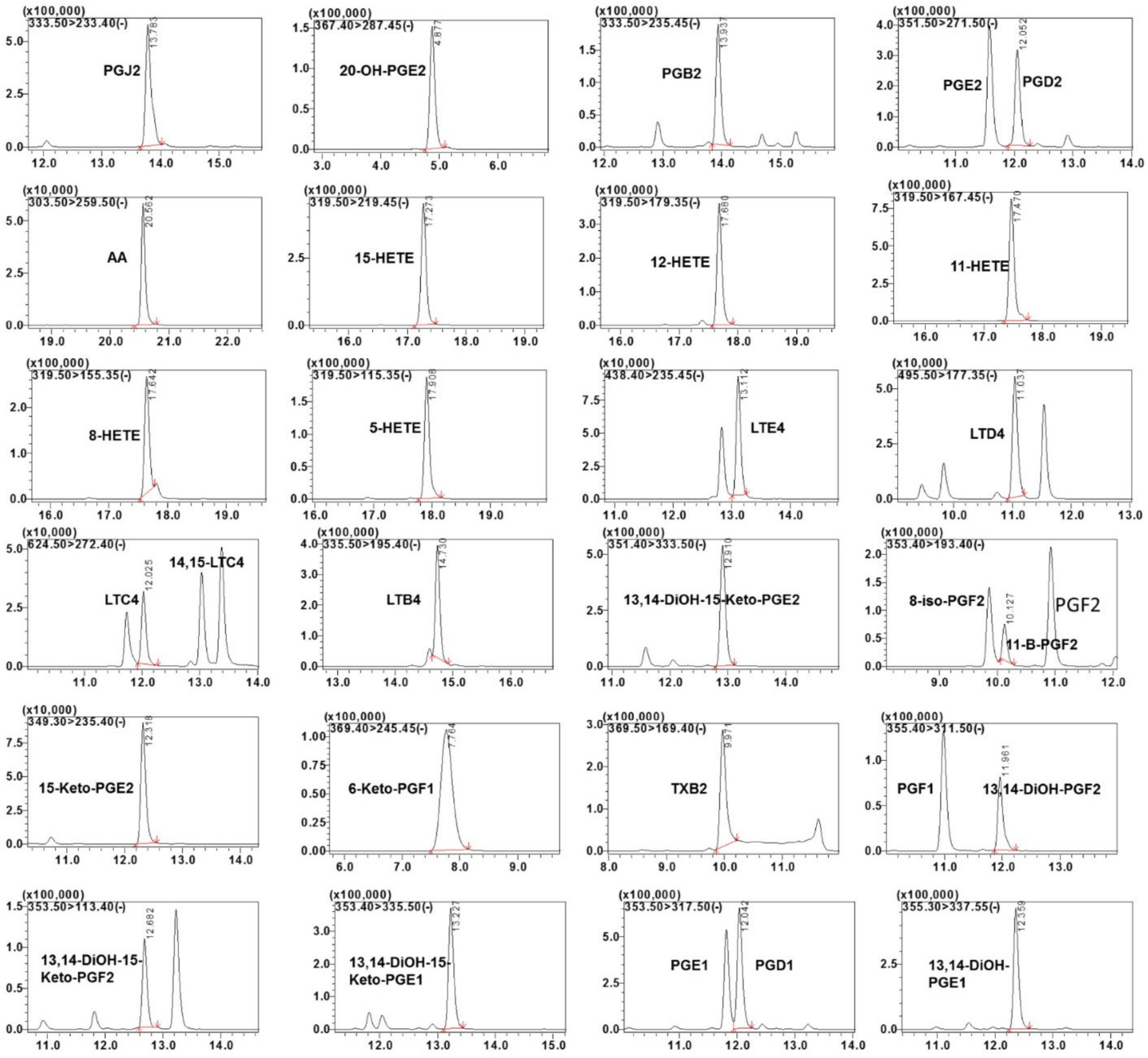

Figure S1. (Continued…)

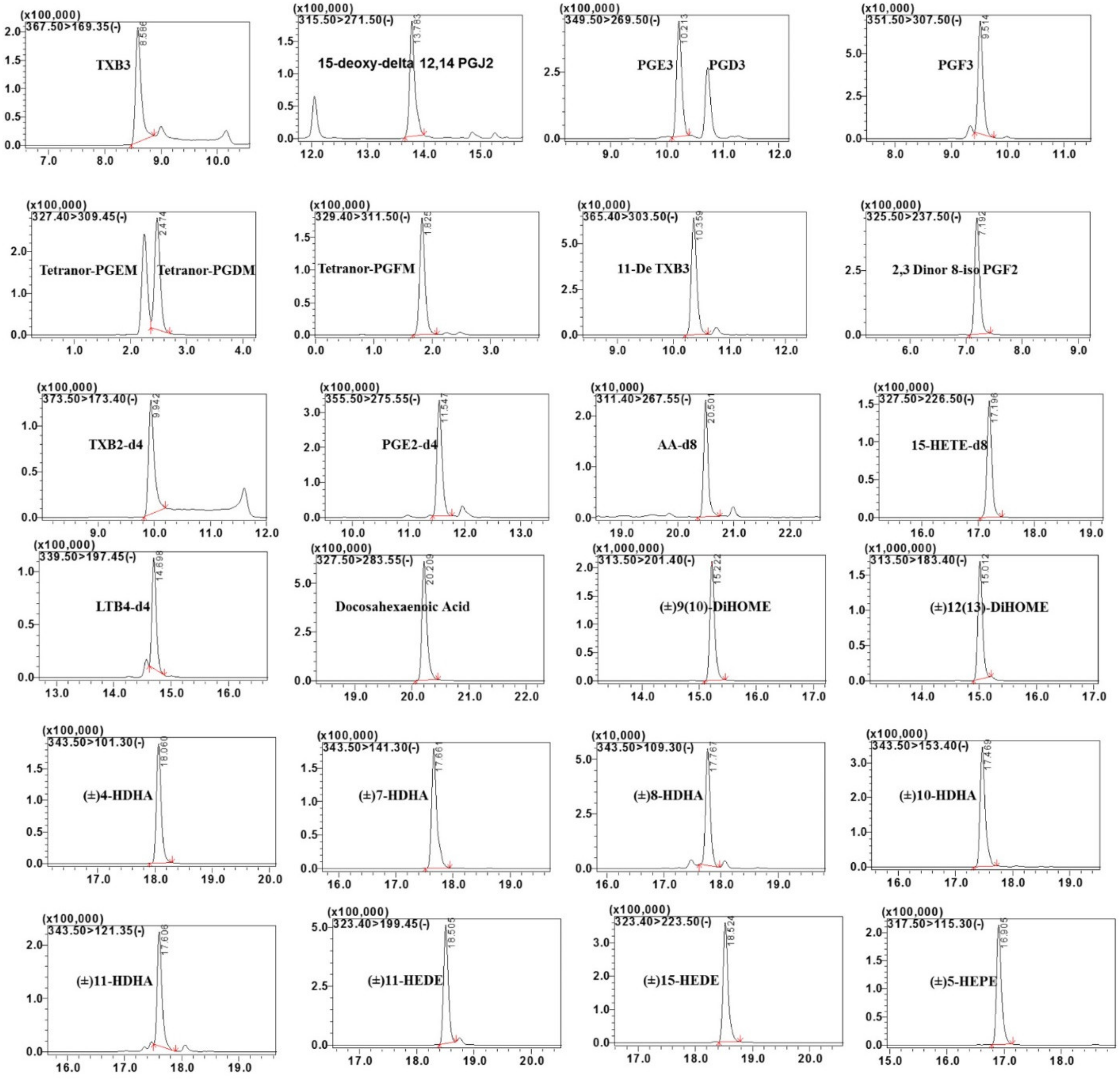
367.50>169.35(-)
TXB3
315.50>271.50(-)
15-deoxy-delta 12,14 PGJ2
349.50>269.50(-)
PGE3
PGD3
351.50>307.50(-)
PGF3
327.40>309.45(-)
Tetranor-PGEM
Tetranor-PGDM
329.40>311.50(-)
Tetranor-PGFM
365.40>303.50(-)
11-De TXB3
325.50>237.50(-)
2,3 Dinor 8-iso PGF2
373.50>173.40(-)
TXB2-d4
355.50>275.55(-)
PGE2-d4
311.40>267.55(-)
AA-d8
327.50>226.50(-)
15-HETE-d8
339.50>197.45(-)
LTB4-d4
327.50>283.55(-)
Docosahexaenoic Acid
313.50>201.40(-)
(±)9(10)-DiHOME
313.50>183.40(-)
(±)12(13)-DiHOME
343.50>101.30(-)
(±)4-HDHA
343.50>141.30(-)
(±)7-HDHA
343.50>109.30(-)
(±)8-HDHA
343.50>153.40(-)
(±)10-HDHA
343.50>121.35(-)
(±)11-HDHA
323.40>199.45(-)
(±)11-HEDE
323.40>223.50(-)
(±)15-HEDE
317.50>115.30(-)
(±)5-HEPE

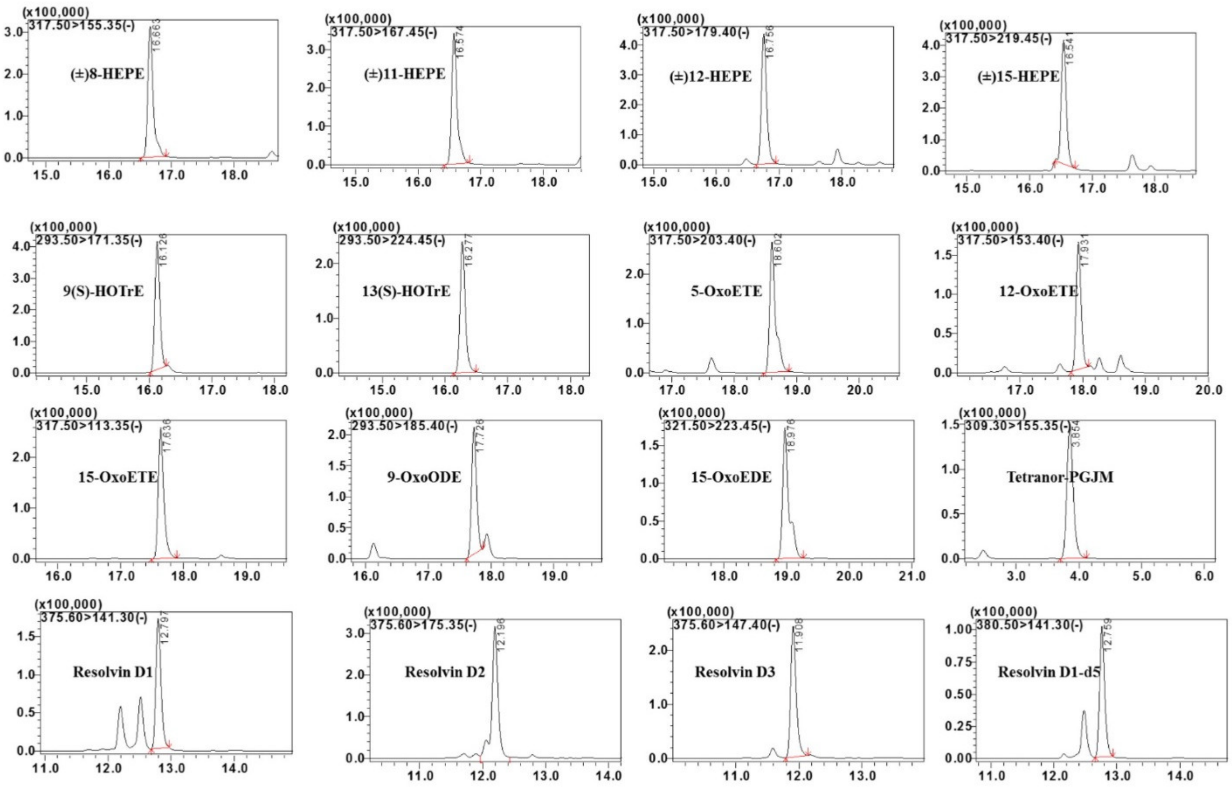
(x100,000)
317.50>155.35(-)
(±)8-HEPE
317.50>167.45(-)
(±)11-HEPE
317.50>179.40(-)
(±)12-HEPE
317.50>219.45(-)
(±)15-HEPE
293.50>171.35(-)
9(S)-HOTrE
293.50>224.45(-)
13(S)-HOTrE
317.50>203.40(-)
5-OxoETE
317.50>153.40(-)
12-OxoETE
317.50>113.35(-)
15-OxoETE
293.50>185.40(-)
9-OxoODE
321.50>223.45(-)
15-OxoEDE
309.30>155.35(-)
Tetranor-PGJM
375.60>141.30(-)
Resolvin D1
375.60>175.35(-)
Resolvin D2
375.60>147.40(-)
Resolvin D3
380.50>141.30(-)
Resolvin D1-d5

References

1. Blewett, A. J.; Varma, D.; Gilles, T.; Libonati, J. R.; Jansen, S. A., Development and validation of a high-performance liquid chromatography-electrospray mass spectrometry method for the simultaneous determination of 23 eicosanoids. *J Pharm Biomed Anal* **2008,** 46, (4), 653-62.
2. Yamada, M.; Kita, Y.; Kohira, T.; Yoshida, K.; Hamano, F.; Tokuoka, S. M.; Shimizu, T., A comprehensive quantification method for eicosanoids and related compounds by using liquid chromatography/mass spectrometry with high speed continuous ionization polarity switching. *Journal of Chromatography B* **2015,** 995–996, 74-84.
3. Wong, A.; Sagar, D. R.; Ortori, C. A.; Kendall, D. A.; Chapman, V.; Barrett, D. A., Simultaneous tissue profiling of eicosanoid and endocannabinoid lipid families in a rat model of osteoarthritis. *Journal of lipid research* **2014,** 55, (9), 1902-1913.
4. Shaik, J. S. B.; Miller, T. M.; Graham, S. H.; Manole, M. D.; Poloyac, S. M., Rapid and simultaneous quantitation of prostanoids by UPLC–MS/MS in rat brain. *Journal of Chromatography B* **2014,** 945, 207-216.
5. Le Faouder, P.; Baillif, V.; Spreadbury, I.; Motta, J.-P.; Rousset, P.; Chêne, G.; Guigné, C.; Tercé, F.; Vanner, S.; Vergnolle, N., LC–MS/MS method for rapid and concomitant quantification of pro-inflammatory and pro-resolving polyunsaturated fatty acid metabolites. *Journal of Chromatography B* **2013,** 932, 123-133.
6. Schoeman, J. C.; Harms, A. C.; van Weeghel, M.; Berger, R.; Vreeken, R. J.; Hankemeier, T., Development and application of a UHPLC-MS/MS metabolomics based comprehensive systemic and tissue-specific screening method for inflammatory, oxidative and nitrosative stress. *Analytical and bioanalytical chemistry* **2018,** 410, (10), 2551-2568.
7. Yue, H.; Jansen, S. A.; Strauss, K. I.; Borenstein, M. R.; Barbe, M. F.; Rossi, L. J.; Murphy, E., A liquid chromatography/mass spectrometric method for simultaneous analysis of arachidonic acid and its endogenous eicosanoid metabolites prostaglandins, dihydroxyeicosatrienoic acids, hydroxyeicosatetraenoic acids, and epoxyeicosatrienoic acids in rat brain tissue. *Journal of pharmaceutical and biomedical analysis* **2007,** 43, (3), 1122-34.
8. Jouvene, C.; Fourmaux, B.; Geloen, A.; Balas, L.; Durand, T.; Lagarde, M.; Letisse, M.; Guichardant, M., Ultra-Performance Liquid Chromatography-Mass Spectrometry Analysis of Free and Esterified Oxygenated Derivatives from Docosahexaenoic Acid in Rat Brain. *Lipids* **2018,** 53, (1), 103-116.
